# The Association Between Doctor–Patient Conflict and Uncertainty Stress During Clinical Internships Among Medical Students: A Panel Study

**DOI:** 10.3390/healthcare13091080

**Published:** 2025-05-06

**Authors:** Huihui Wang, Xinxin Ying, Lujin Zhang, Tingzhong Yang, Weifang Zhang

**Affiliations:** 1 Stomatology Hospital, School of Stomatology, Zhejiang University School of Medicine, Hangzhou 310006, China; huihui_wang@zju.edu.cn (H.W.); zhanglj77@zju.edu.cn (L.Z.); 2 Clinical Research Center for Oral Diseases, Key Laboratory of Oral Biomedical Research of Zhejiang Province, Hangzhou 310006, China; 3 Cancer Center of Zhejiang University, Engineering Research Center of Oral Biomaterials and Devices of Zhejiang Province, Hangzhou 310006, China; 4 Women’s Hospital, School of Medicine, Zhejiang University, Hangzhou 310006, China; yxinxin@zju.edu.cn; 5 Center for Tobacco Control Research, School of Medicine, Zhejiang University, Hangzhou 310058, China; 6 Injury Control Research Center, West Virginia University, Morgantown, WV 26506, USA

**Keywords:** medical students, clinical internship, uncertainty stress, doctor–patient conflict, reference norm, longitudinal study

## Abstract

Background/Objectives: Medical students experience significant mental stress during clinical internships. This study aimed to assess the levels of uncertainty stress among medical interns, evaluate its temporal changes and associations with doctor–patient conflict and reference norm, and provide insights for stress-alleviating policies and educational initiatives. Methods: A prospective longitudinal panel study was conducted; 131 medical students preparing for clinical internships were recruited via WeChat social media groups from June 2023 to June 2024. Data were collected at three time points using an online survey on Wenjuanxing: before the internship, three months into the internship, and after the internship. Variables such as uncertainty stress, doctor–patient conflict, and reference norm were measured, and data were analyzed using GEE and the GLMM program. Results: A total of 122 students completed all three waves of the study. Uncertainty stress decreased over the internship period (W = 7.25, *p* < 0.05), while doctor–patient conflict increased (W = 6.65, *p* < 0.05). Uncertainty stress was positively associated with the reference norm from teachers (β = 0.856, *p* < 0.05) and doctor–patient conflict (β = 1.068, *p* < 0.05). Conclusions: Although uncertainty stress reduces as internships progress, doctor–patient conflict rises. A supportive learning environment, especially from teachers, is crucial for mitigating stress. Medical schools and hospitals should implement comprehensive strategies to address individual stressors and institutional factors, considering the associations between uncertainty stress, doctor–patient conflict, and reference norm. However, the study has limitations such as a small sample size and reliance on self-reported measures, indicating a need for further research.

## 1. Introduction

Mental stress refers to the strain or threat posed by various adverse events or circumstances from daily life and workplace, which manifest as both physical and mental tension and discomfort [1,2]. Research indicates that severe mental stress can lead to a wide range of social and health problems, particularly psychological disorders and behavioral issues [3,4]. Globally, medical education systems share similarities in exposing students to high-stress environments. For instance, studies in North America highlight that medical students report clinically significant stress levels during clinical rotations, with burnout rates exceeding 50% in some cohorts [5,6]. Similarly, research from Poland emphasizes the compounding effects of academic demands and peer pressure on trainees’ mental health [7].

In China, the medical education system typically consists of a five-year undergraduate program, followed by potential postgraduate training. During the undergraduate program, students spend the initial years (generally the first three) focusing on theoretical knowledge, basic medical sciences, and preclinical courses. The clinical internship, a critical phase of practical training, generally begins in the fourth or fifth year of the program, depending on the institution [3]. Clinical internship, an essential component of medical education, plays a vital role in cultivating students’ clinical capabilities and professional ethics [8]. This is of great significance for training qualified doctors. Typically, the clinical internship is arranged in the final year or two of undergraduate education.

Medical students face numerous challenges in the process of their academic and career development. They not only need to cope with heavy academic courses and take on important responsibilities in clinical practice but they also have to confront the uncertainties in their future career development [9]. This phenomenon is not unique to China. A cross-sectional study revealed that 55.4% of medical interns experienced “extreme stress” due to career uncertainty in North India [10]. A number of studies have highlighted that medical students experience significant mental stress, which necessitates further attention and intervention [9].

The stress is particularly prevalent during the clinical internship period for medical students [11]. After completing four years’ theoretical study, medical students face a one-year internship, transitioning from a familiar campus environment to the complex, high-pressure atmosphere of a hospital [12]. This shift involves significant personal and professional changes, including navigating the intricate dynamics of the doctor–patient relationship, preparing for postgraduate exams, and addressing the concerns of career decision and employment competition [13]. International perspectives underscore the universality of these challenges. A Korean study identified “role transition stress” as a key predictor of anxiety among medical interns, particularly when interacting with patients independently for the first time [14]. Meanwhile, US research demonstrates that structured Peer-Mentorship programs can mitigate stress, highlighting potential intervention pathways [15].

The clinical internship is a pivotal period for medical students; during the clinical internship, students transition from a relatively familiar learning environment to an unfamiliar one, facing unfamiliar clinical duties, ambiguous role expectations, and complex interpersonal dynamics with supervisors, peers, and patients, during which they frequently encounter substantial mental stress [9]. This stress can evoke intense emotional responses. Based on Festinger’s cognitive dissonance theory, uncertainty has the potential to induce cognitive chaos [16]. When individuals are confronted with uncertainty, they may experience confusion in their cognitive processes, which often prompts them to adopt avoidance behaviors [17,18]. These avoidance behaviors, in turn, exacerbate the stress levels. Uncertainty-related stress is notoriously challenging to manage and can have significant negative implications [19].

During their internships, medical students are faced with a multitude of challenges [20]. They are thrust into an unfamiliar hospital environment, tasked with new job responsibilities, and burdened by concerns about their future employment [21]. Among these challenges, navigating the intricate doctor–patient relationships is particularly daunting as they have little to no prior experience in such situations [22,23]. All these factors contribute to the development of high levels of uncertainty stress.

This research is centered around the problem of uncertainty stress among clinical interns. Its objectives are three-fold: to measure the degree of uncertainty stress that medical students experience during their clinical internships; to monitor the temporal variations in uncertainty stress, doctor–patient conflicts, and the opinions of relevant reference groups throughout the internship; and to explore the correlations between uncertainty stress, doctor–patient conflicts, and the views of relevant reference groups. The findings of this study are expected to offer valuable insights for the formulation of policies and educational initiatives aimed at alleviating the mental stress of medical students, thereby facilitating their well-being and professional development.

## 2. Materials and Methods

### 2.1. Study Design

A prospective longitudinal panel study was designed to explore the temporal trends of uncertainty stress, doctor–patient conflict, and the views of relevant reference norms during clinical internships.

Participants were sourced through advertisements disseminated in social media groups on WeChat, a prevalent platform in China. The inclusion criteria were as follows: being medical students about to commence their clinical internships, having access to a smartphone, being proficient in Chinese, and demonstrating a willingness to engage in the study and provide follow-up information. The exclusion criteria encompassed a refusal to provide essential data or having any medical conditions that might restrict participation. After providing consent, participants would receive an online questionnaire with instructions; participants were requested to confirm the “Confirmation and Authorization” before proceeding to the survey. Informed consent was obtained by an independent research assistant unaffiliated with the participants’ academic departments. No investigators involved in the study held teaching or evaluative roles involving the participants during the data collection period. Consent forms explicitly stated that participation was voluntary and would not impact academic standing. All data were anonymized. A dedicated WeChat group was created to manage the follow-up data collection, with each participant receiving a unique QR code to prevent unauthorized responses and ensure traceability.

Data were collected over three phases: Phase 1 (before the clinical internship), Phase 2 (after three months into the clinical internship), and Phase 3 (after the internship). The data collection period spanned from June 2023 to June 2024.

### 2.2. Data Collection

The survey was conducted with an online tool called Wenjuanxing (www.wjx.cn) from 1 June 2023 to 1 June 2024, a popular Chinese survey platform similar to Qualtrics or SurveyMonkey. A unique link was provided for each phase of the study, and participants accessed the survey through the WeChat group. All responses were anonymous, and the survey took approximately 10 min to complete. The same protocol was followed across all phases to ensure consistency in data collection.

### 2.3. Measurement

Demographic variables such as age, year, gender, ethnicity, residence, and parental education levels were recorded. Doctor–patient conflict was assessed as the primary explanatory variable, using the question “Have you experienced any unpleasant incidents between doctors and patients in the department during your study/internship?”. The key variable of this study composition is based on the Risk Belief and Subjective Norm Theory. The reference norm reflects students’ perceptions of the opinions about the medical profession, in general, held by individuals with whom they share close relationships, including family members, relatives, friends, classmates, and teachers, specifically regarding doctors. Responses were measured using a 5-point Likert scale ranging from ‘very good’ to ‘very poor’ (with higher scores indicating poorer perceived opinions).

Uncertainty stress was measured by the 4-item Chinese Uncertainty Stress questionnaire, which was developed by Dr Yang [24], without modifications to item wording, structure, or contents. This instrument also has demonstrated acceptable reliability with Cronbach’s alpha values for the USS across waves (baseline: 0.875; 3-month follow-up: 0.901; and end of internship: 0.886). It covered 4 items: current life uncertainty (“Life is unpredictable and beyond our control ”); social change uncertainty (“The world is changing too fast, and I can’t keep up with it”); goals uncertainty (“I don’t know how to achieve my goals”); and social values uncertainty (“Social values are in chaos, and I don’t know what to do”). Respondents rated these items on a 5-point scale from feeling no stress (0), a little stress (1), some stress (2), considerable stress (3), and very strong stress (4). A total stress score was obtained by summing the responses to the individual questions. The higher the total score, the greater the perceived level of stress [25,26].

### 2.4. Data Analysis

Data were recorded into Microsoft Excel and imported into SPSS (version 25.0) for statistical analysis. GEE was used to evaluate the temporal trends in uncertainty stress and related variables, Post hoc tests were conducted to examine differences between time points, utilizing Tukey-corrected pairwise tests. The generalized linear mixed model (GLMM) was used to examine associations between uncertainty stress and related variables. The models included participant-specific random intercepts to handle within-subject correlations. We specified a binomial distribution with logit link function for our outcome variable. Time was modeled as a continuous fixed effect, with potential nonlinear trends examined through quadratic terms. The fixed effects structure incorporated doctor–patient conflict, reference norm from family or relatives, reference norm from friends, reference norm from classmates, and reference norm from teachers. The reported β coefficients primarily reflect within-subject effects, unless otherwise noted in the results. All statistical models were evaluated for their respective assumptions. For GEE models, we verified the appropriateness of the working correlation structure (exchangeable) using quasi-likelihood information criterion (QIC). GLMM assumptions were checked by examining residual distributions and random effects normality plots. When model assumptions were not fully met, we applied robust standard errors (GEE) or considered alternative distributions (GLMM).

## 3. Results

A total of 131 participants were recruited at baseline, with 122 (93%) completing all three waves of the study. Among all participants, 52.5% of them were females. In addition, most of the participants were Han Chinese, at 99.2%. In terms of residence, 37.7% of participants come from rural areas, while the percentage of participants coming from urban areas, counties, and townships were lower, at 27.9%, 19.7%, and 14.8%. Regarding parental education, 14.8% of participants’ fathers and 9.8% participants’ mothers had a bachelor degree or higher education (Table 1).

**Figure 1:**
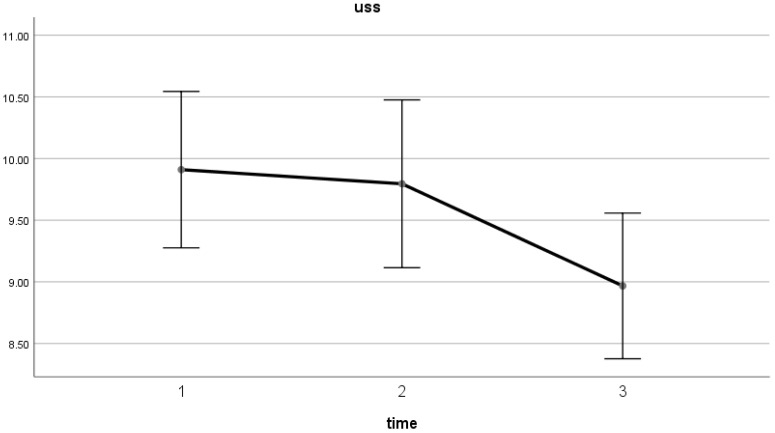
Uncertainty stress changes across the three phases.

The results indicate significant changes in uncertainty stress over the course of the study (W = 7.25, *p* < 0.05, Figure 1). It is the proportion reporting a doctor–patient conflict that experienced increased uncertainty stress (W = 6.65, *p* < 0.05). Similarly, there was an increase in the reference norm from teachers (higher scores indicating poorer perceived opinions) (Table 2).

In Table 3, our generalized linear mixed model (GLMM) analysis revealed significant positive associations between uncertainty stress and doctor–patient conflict (β = 1.068, SE = 0.525, *p* < 0.05), with an odds ratio of 2.91. This represents a large effect sizesuggesting that each unit increase in doctor–patient conflict nearly triples the odds of experiencing uncertainty stress. Similarly, the reference norm from teachers showed a significant positive association (β = 0.856, SE = 0.373, *p* < 0.05), corresponding to an odds ratio of 2.35, indicating a medium-to-large effect size. These substantial effect magnitudes suggest that both doctor–patient conflict and reference norms from teachers have clinically meaningful impacts on uncertainty stress.

## 4. Discussion

This study fills a gap in the existing literature by examining how uncertainty stress evolves over time among clinical interns. Previous research has not fully explored the temporal changes in this specific stressor. The findings highlight a significant reduction in uncertainty stress as the internship progresses. The average stress score decreased from 9.91 at the start of the internship to 8.97 at the final observation point, with the highest recorded score at baseline.

Medical students typically undergo a one-year clinical internship after completing four years of theoretical coursework. This transition marks a significant shift from the familiar and controlled campus environment to the unpredictable and complex hospital setting. Along with adjusting to new clinical duties, students also face various external pressures, such as navigating complex doctor–patient relationships, preparing for postgraduate entrance exams, making career decisions, and dealing with employment concerns [26]. As a result, the internship period is often marked by high levels of psychological stress and intense emotional experiences [27]. Over the course of the internship, as medical students gradually become more acquainted with the clinical environment, the stress associated with uncertainty shows a tendency to decline. This decrease in stress indicates that interns develop greater adaptability and resilience as they accumulate more experience. At the beginning, the stress brought about by an unfamiliar environment and complex responsibilities can be extremely burdensome [28]. However, as students become more self-assured in their abilities and devise effective coping mechanisms, they are better equipped to handle uncertain situations [29]. Their growing familiarity with the hospital environment, job duties, and the nuances of doctor–patient interactions enables them to build up resilience, which eventually results in a reduction in uncertainty-related stress during the entire internship [30].

Previous research has emphasized a significant link between doctor–patient conflict and uncertainty stress [31]. The Stimulus, Cognition, and Response (SCR) theory posits that environmental stimuli, regardless of whether an individual experiences them directly or not, can trigger both physiological and psychological reactions by affecting their cognitive system [32]. As interns progress in their clinical work, they become more acutely aware of the pressure related to uncertainty, and this leads to a better understanding of its complex nature [33]. The findings of this study emphasize the importance of behavioral interventions grounded in perceived beliefs. These interventions are designed to offer individuals opportunities to recognize and confront the challenges they encounter. By addressing the underlying cognitive structures, these interventions can promote behavioral change [34].

Moreover, research has shown that experiences of doctor–patient conflict and the attitudes of supervising physicians are strongly associated with mental stress. From a normative perspective, individuals who have significant relationships with medical students, such as mentors and senior physicians, play a critical role in shaping their stress levels [35]. This dynamic is reflected in the concept of the “neighborhood effect” in behavioral studies, where social interactions and environmental factors influence individual behavior. Consequently, when it comes to stress-reduction interventions, it is essential to not merely concentrate on individual interns. Instead, strategies at the group level should be integrated [36]. This might involve fostering positive peer influence and leveraging “opinion leaders” to advocate for positive behavioral shifts [37].

The outcomes of this research offer valuable perspectives on the trends of uncertainty stress and its relationship with risk beliefs and subjective norms among clinical interns. These insights present educational and administrative departments with a novel vantage point, enabling them to gain a better understanding of the uncertainty stress that medical students undergo and manage it more effectively. The study results indicated a decline in uncertainty stress during the observation period. However, it is noteworthy that negative perceptions from the teachers increased during the study period, suggesting an escalation in industry-related stressors during the clinical internship. Previous research has shown that excessive uncertainty stress can negatively impact work efficiency, increase the possibility of errors, accidents, as well as adverse health outcomes [38]. This underscores the importance of educational management departments monitoring uncertainty stress and tailoring educational interventions in a timely manner to help medical students manage excessive pressure.

Furthermore, our study found that perceived beliefs and social norms are closely linked to the uncertainty stress of medical students. Stress is an inevitable aspect of modern life, but minimizing its harmful effects is crucial. One effective strategy is to control the sources of stress. Medical students are inherently part of a high-stress cohort, and it is essential for management departments to regulate assessment criteria and stress levels within manageable bounds. Additionally, the study revealed that the influence of reference norms contributes to uncertainty stress among medical interns. This social issue, characterized by negative perceptions of doctors within the medical community, further exacerbates stress. Medical schools and hospitals must place a strong emphasize on medical ethics and professional conduct. Simultaneously, efforts should be made to combat societal misconceptions about the medical profession by promoting a positive public image of doctors.

### Limitations and Future Directions

While this study provides valuable insights into the relationship between uncertainty stress and doctor–patient conflict, there are several limitations. Firstly, the sample size is relatively small, and therefore the results may not be fully generalizable. Future studies should include larger, more diverse samples to enhance the robustness of the findings. The use of convenience sampling, though practical for logistical constraints, may have introduced selection bias, which limits the generalizability of findings to broader populations with differing socio-cultural or institutional contexts. Future studies should employ stratified or randomized sampling techniques to enhance representativeness. Secondly, the study only used self-reported measures of uncertainty stress, doctor–patient conflict, and reference norms which may be subject to response biases. Future research should consider including objective measures of stress and conflict, such as physiological indicators or observational data. Thirdly, the study relied on single-item scales to measure some variables, which may not fully capture the complexity of the constructs, we will use validated, multi-item instruments in future research to improve reliability and depth. Future studies should use multi-item scales to assess these variables more comprehensively. Fourthly, the sample is predominantly Han Chinese and drawn from a single region via social media recruitment, which may introduce selection bias and limit external validity. Finally, the study’s longitudinal design allows for the examination of temporal trends, and further research might benefit from incorporating more frequent data collection points to capture more nuanced changes over time. Additionally, investigating the long-term effects of uncertainty stress beyond the internship period would provide a more comprehensive understanding of how these experiences influence medical students’ mental health and professional development.

## 5. Conclusions

This study contributes to the growing body of research on uncertainty stress and its effects on medical students during clinical internships. The findings suggest that while uncertainty stress decreases over time, doctor–patient conflict increase as students progress through their internships. The associations between uncertainty stress and reference norms from teachers underscore the importance of a supportive learning environment in mitigating stress. To address both individual stressors and institutional factors, and to improve medical students’ well-being and professional development during the internships, medical schools and hospitals must implement comprehensive strategies to support them. The results of this study have significant implications for medical education, offering insights into the psychological pressures faced by medical students and providing a basis for interventions aimed at reducing uncertainty stress and fostering better clinical training environments.

## Figures and Tables

**Table 1:** Demographic characteristics of participants.

Group	N	Percentage
**Gender**		
Male	58	47.5
Female	64	52.5
**Ethnicity**		
Han	121	99.2
Minority	1	0.8
**Residence**		
Rural	46	37.7
Township	18	14.8
County	24	19.7
Urban	34	27.9
**Year**		
Grade Four	63	51.6
Grade Five	59	48.4
**Grades**		
Upper-third	36	29.5
Middle-third	51	41.8
Lower-third	35	28.7
**Father’s educational level**		
Elementary school and below	11	9.0
Junior high school	41	33.6
High school	29	23.8
College diploma	23	18.9
Bachelor’s degree or above	18	14.8
**Mother’s educational level**		
Elementary school and below	15	12.3
Junior high school	46	37.7
High school	28	23.0
College diploma	21	17.2
Bachelor’s degree or above	12	9.8

**Table 2:** Time change trend in uncertainty stress, doctor–patient conflict, and reference norm (mean, SD).

Group	N	Uncertainty Stress	Doctor–Patient Conflict	Reference Norm from Family or Relatives	Reference Norm from Friends	Reference Norm from Classmates	Reference Norm from Teachers
Time1	122	9.91 (0.32) ^a^	^a^	1.78 (0.07)	1.80 (0.06)	1.89 (0.07)	1.81 (0.07) ^a^
Time2	122	9.80 (0.34) ^a^	^b^	1.88 (0.06)	1.90 (0.06)	2.02 (0.07)	1.99 (0.07) ^b^
Time3	122	8.97 (0.30) ^b^	^b^	1.73 (0.06)	1.80 (0.06)	1.93 (0.07)	2.01 (0.07) ^b^
Wald (p)		7.25 (0.03)	6.65 (0.04)	2.25 (0.10)	3.29 (0.19)	3.33 (0.19)	7.06 (0.03)

Note: ^a^ and ^b^ mean data in the same column with the same superscript letter indicate that there is no significant difference (*p* > 0.05), while data with different superscript letters indicate a significant difference (*p* < 0.05).

**Table 3:** Association between doctor–patient conflict and uncertainty stress.

	β	SE	t	*p*
uncertainty stress → doctor–patient conflict	1.068	0.525	2.036	0.042 *
uncertainty stress → reference norm from family or relatives	0.503	0.463	1.086	0.278
uncertainty stress → reference norm from friends	−0.888	0.520	−1.078	0.088
uncertainty stress → reference norm from classmates	0.229	0.408	0.561	0.575
uncertainty stress → reference norm from teachers	0.856	0.373	2.295	0.022 *

* indicate that the difference is statistically significant.

## Data Availability

The data that support the findings of this study are available from the corresponding author upon reasonable request. Due to privacy concerns regarding the medical students’ personal information and responses, the data are not publicly available. However, researchers interested in further analyzing the data can contact the corresponding author to discuss access and data-sharing arrangements in line with ethical and privacy considerations.
